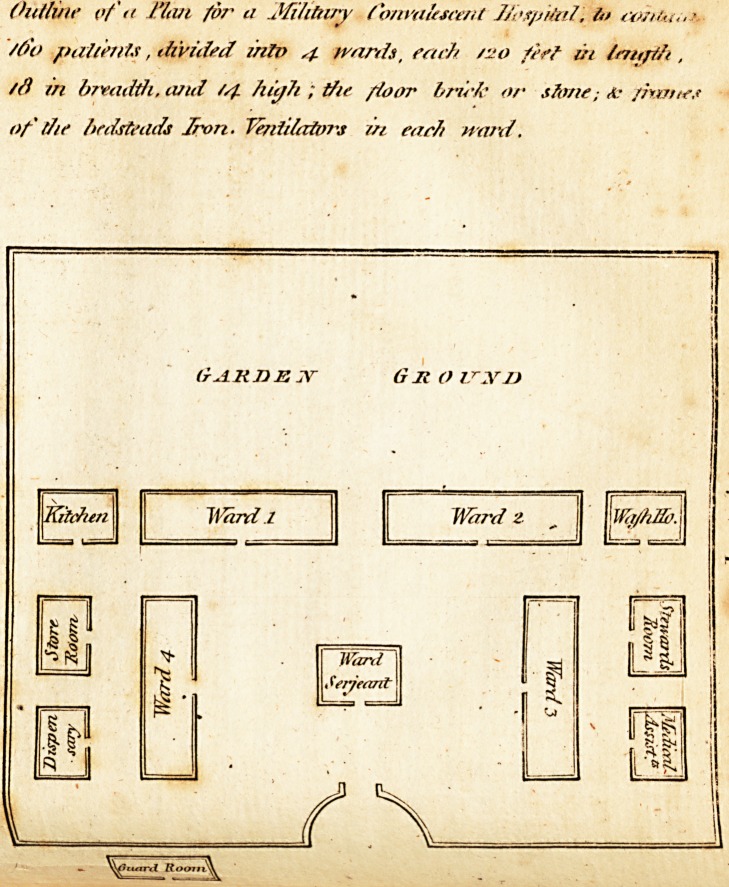# An Inquiry into the Causes Which Produce Disease among the Troops at the Cape of Good Hope, with a View to Discover the Most Effectual Means of Prevention;—To Which Is Added, the Outline of a Plan of Military Hospitals, on a Principle and Construction Tending to Introduce a More Successful Treatment of the Sick

**Published:** 1799-07

**Authors:** Stewart Henderson

**Affiliations:** late Apothecary to His Majesty's Forces at the Cape


### *An Inquiry into the Causes which produce Disease among the Troops at the Cape of Good Hope, with a view to discover the most effectual Means of Prevention;—to which is added, the Outline of a Plan of Military Hospitals, on a principle and construction tending to introduce a more successful treatment of the Sick.*

[By Stewart Henderson, late Apothecary to His Majesty's Forces at the Cape.]

THE following investigation was suggested in consequence of His Majesty's troops being for some time very sickly in this quarter, and erroneous opinions having gone abroad, respecting the cause of the sickness and mortality which ensued:

The plan of military hospitals, of which I here give the outline, is on a principle and construction different, I believe, from any yet established in Europe.

The want of proper military hospitals has been severely felt, I may say, both at home and abroad; but since his Royal Highness the Duke of York filled the important station of Commander in Chief, those useful national institutions, which reflect so much honour on a country, are erecting, I understand, in different parts of the kingdom.

In the latter end of 1793, a regiment arrived at Plymouth, from Cork, in a very sickly state, having upwards of three hundred men ill of a malignant jail-fever. The best, and only accommodation that could be had, was a large stable, ill calculated to contain such a number of men, labouring under infectious disease, and in a crouded situation. If proper ventilation, and hospital-necessaries cannot be procured, medical efforts avail but little. As might be expected, every symptom was aggravated, and the effect of medicine counteracted. From what I have observed in the four quarters of the world, where I have been employed on actual service, in time of war, the places allotted for, and named hospitals, appeared to me only adapted for men to die in, not to recover from disease; nor do I think that the great sickness and mortality which have happened to the British troops, in different parts, particularly in the West Indies, and on the Continent, have actually arisen from the irremediable effect of climate, or unavoidable hardships of service, but from causes which I think might have been greatly obviated, if not entirely prevented: it is therefore to be hoped, that we shall profit from experience experience and paſt misfortunes, by adopting thoſe means of prevention, which I am well convinced are within our power, requiring only the aid and energy of the legiſlature, to appoint proper officers of health, eſtabliſh lazarettos, with the obſervance of quarantine, which are found ſo neceſſary in other countries for preventing the introduction of infectious diſeaſe, as well as having hoſpitals conſtructed on a proper principle; and that the medical department in theſe countries be filled by men of obſervation and experience.

Had theſe precautions been adopted, and attended to in the Weſt Indies, in my opinion, the ſaving to the nation, in men and money, would have been immenſe. We can only lament, that they were not put in practice; as they would, in all probability, have effectually deſtroyed the contagion at Grenada, and prevented the importation of that peſtilence into the other iſlands, in the manner related by Dr. Chisholm, in his book on the Yellow Fever.

I ſubmitted a plan to government in 1791 (which I ſhall inſert at the end of the following pages), for preventing the introduction of infectious diſeaſe, into the garriſon at Chatham, which was approved of by the Surgeon-general, Mr. John Hunter, who ſtrongly recommended the adoption of it. I was afterwards informed, that it had been adopted by the Secretary at War, and found not to anſwer. To be ſatisfied on this point, I went to Chatham, and found that a ſhip indeed had been taken up, but not appropriated for the purpoſe of prevention (the important object I had in view), but for the reception of ſick, of different complaints, as well as priſoners. General Fox, the commanding officer of the garriſon, at the ſame time informed me in writing, that he would never be induced to recommend ſoldiers of any deſcription to be put on board ſhip, except ſuch as were priſoners, merely for the ſake of ſecurity, when there were not ſufficient priſons on ſhore.

As there is no building unconnected with the garriſon, for the purpoſe of prevention, and the hoſpital being a part of the barracks, it is evident, from the deſcription of men brought in, that infection muſt be, I may ſay, daily introduced, and, I much fear, a portion of it exported to different parts of the world, by every detachment ſent from thence, notwithſtanding the prudent and judicious meaſures ordered to be purſued by the commanding officer, as well as the attention paid by the gentleman who ſuperintends the medical department—but medical men can do no more than recommend. However, ſince the publication of my printed letter, in January 1795 (addreſſed to the officers of the army under orders for the Weſt Indies), on the means of preſerving health, I find that certain articles of diet and neceſſaries for the ſick, which I particularly recommended in that letter, are now ſupplied at the the expence of government, for the uſe of the troops on board; and I can affirm, that the moſt beneficial and ſalutary effects have ſince been experienced, by the arrival of ſome regiments at the Cape from England, without having any ſick; the conſequence, no doubt, of prevention being better underſtood and attended to.

Travellers who have viſited this part of Africa, for the purpoſe of inveſtigating the Natural Hiſtory of the country, ſpeak with rapture of the ſalubrity of its air, and natural productions. I think it will be found, upon inquiry, to merit the moſt favourable opinion. The face of the country, for a conſiderable diſtance (except the land which forms the Cape), is rather low, but open, and well cultivated, of a ſandy and marly ſoil, which ſoon abſorbs the rain, thereby preventing the noxious exhalations, ſo productive of ſickneſs in hot climates. The air, except a few months, may be called temperate; the atmoſphere is ſeldom loaded with moiſture, but poſſeſſes a degree of elaſticity not often felt in any other country; and although Fahrenheit's thermometer, during the ſummer months*, ranges from 80 to 90, and frequently a conſiderable variation is ſuddenly experienced, yet from the dryneſs of the atmoſpheric air, and a briſk circulation of it being kept up by the prevailing South Eaſt Winds, the conſtitution ſuffers little from relaxation. Invalids from India, labouring under complaints of debility, the effect of great heat, ſoon recover their ſtrength here, by the temperate and bracing air of the winter months; and a further proof of its ſalubrity, are the florid and healthy looks we perceive in the Dutch inhabitants, eſpecially thoſe who live in the country, and are not enervated by luxury and indolence; though it is remarked that there are not many inſtances of longevity among them, owing in a great meaſure to their habits and manner of life, yet they are happily exempt from many of thoſe endemic and epidemic diſeaſes which rage in other parts of the world, and annually carry off great numbers. The ſmall-pox, meaſles, remittent and intermittent fever, and that moſt fatal of all diſeaſes, the jail, hoſpital, or ſhip-fever, which deſtroys ſo many of the human ſpecies in every part of Europe, are never generated here, and are unknown, but when introduced; which, unfortunately for the natives, has ſometimes happened. We likewiſe find, that neither the inhabitants or officers are attacked with the diſeaſes which prevail among the ſoldiers; and it is a ſingular circumſtance, that not an officer of the army or navy has died of diſeaſe contracted here, ſince the Britiſh forces arrived at the Cape (during a period of three years); which I think clearly proves that no noxious quality exiſts in the air of this country,

* November, December, January, and February.

country, which has been by ſome imagined, and erroneouſly blamed, as the cauſe of the malignity of the diſorders, and the many deaths that have occurred in the General Hoſpital. We muſt therefore look for other cauſes than thoſe aſſigned.

The natural productions for the uſe of man perhaps exceed in variety moſt parts of the world. At that ſeaſon of the year when great heat prevails, nature has made ample proviſion to leſſen its influence on the human body, by the abundance of ſweet acid fruits (European and Tropical), of which inſtinct and our reaſon dictate the uſe.

Upon the whole, conſidering its ſituation, climate, and natural productions, ſo far from being deemed unhealthy, it may more properly, in my opinion, be ſtiled the Montpellier of the Southern hemiſphere.

The diſeaſes which prevail among the ſoldiers are, fever, dyſentery, and ulcers.—The principal cauſes ſeem to be the unlimitted and immoderate uſe of ardent ſpirits, want of proper diet, clothing, and bedding.

There is, no doubt, in the human body, a conſtant tendency to putreſcency, more eſpecially in a hot climate, producing various morbid appearances; and this tendency will be increaſed in proportion to the nature and quality of our food. Men uſing much animal food, without a due proportion of vegetable, ſoon contract diſeaſes of a putrid nature; and when aſſiſted by that powerful agent, and deſtructive poiſon, new brandy, which the ſoldiers have ſo much acceſs to, cannot fail to produce ſuch changes, both in the ſolids and fluids, as to aggravate thoſe diſeaſes which, from other cauſes, have incidentally come on.

In a conſtitution thus previouſly prepared, it is eaſy to conceive, that the ſmalleſt ſcratch or wound will degenerate into an ulcer; and if ſeized with fever or dyſentery, the worſt termination may be expected.

I therefore conſider the immoderate uſe of ardent ſpirits to be the great leading cauſe not only of diſeaſe, but thoſe frequent puniſhments which have proved fatal to many of the ſoldiers, from the bad ſtate of their conſtitutions, and foul air of the hoſpitals. In my printed letter, already mentioned, I gave an inſtance of what happened in the ſouthern provinces of India, when there was no arrack for the troops in camp. The ſick conſiderably decreaſed, although the fatigue of duty was great, and the ſeaſon unfavourable; but a few days after, receiving a ſupply of that liquor from Calicut, the ſick-liſt again returned to its uſual ſtandard.

The want of flannel waiſtcoats and bedding in barracks has aſſiſted in producing diſeaſe, particularly the dyſentery. The fever is ſuppoſed to have been introduced from the ſhips, but may likewiſe be generated in the barracks, unleſs the greateſt care is taken to preſerve cleanlineſs and ventilation.

Having endeavoured to ſhow, what are the cauſes of diſeaſe among the troops, it is next to be conſidered in what manner thoſe evils may be obviated.

Many difficulties ariſe on this head, which, perhaps, can only be removed by the ſtrenuous ſupport and exertions of commanding officers. The thoughtleſs ſet of men which the generality of ſoldiers are found to be, with reſpect to what concerns their health, ſhould make them be conſidered and treated as children; obliging them to conform to thoſe regulations, which have been found to be moſt ſalutary, and preclude them as much as poſſible from obtaining what is pernicious.

Cleanlineſs, not only of their perſons, but in and about the barracks, ought to be the firſt conſideration: all filth, dirt, and offal of animals (of which the Cape ſo much abounds), ſhould not be permitted to be left on the ground, but thrown into a pit, and covered with earth.

The barrack-rooms ſhould be frequently white-waſhed, and no obſtruction remain to prevent the free circulation of air.

To guard againſt the variation of the temperature of the atmoſphere, and chill of the night, the men ſhould be obliged to wear a flannel waiſtcoat next their ſkin, which ought to be frequently changed.

They ſhould have bedding, and ſleep in hammocks, or on cott-frames, at leaſt two feet above the ſurface of the floor.

They ſhould have two regular meals a day.—For breakfaſt, tea, coffee, or rice-gruel, whichever can be had at the leaſt expence; and as they have no beer, as in barracks in England, this might be granted in lieu thereof.—In the Weſt Indies, and other climates, a hot breakfaſt has been found moſt conducive to heath. For dinner, beef, or mutton, made into ſoup, with a proportion of vegetables, and ſeaſoned with capſicum, the pepper of the country: a cabbage would alſo be daily neceſſary for every ſix men; but as ſoldiers' pay may not be adequate to purchaſe a ſufficiency, might not the public grounds, or waſte land, be cultivated, for the raiſing of vegetables for the uſe of the ſoldiers?—or it might become an object of government to make ſome allowance for that ſalutary purpoſe; which trifling expence, I am convinced, would in the end be greatly over-balanced, by a conſiderable reduction of hoſpital-expences, and the preſervation of many lives.*

By

* Since this inquiry was drawn up, the soldiers have had their pay increased, by the recommendation of his Royal Highness the Commander in Chief; in consequence of

By attention to a proper diet, their conftitutions would be more able to refift the baneful effects of that deftructive poifon before alluded to (new brandy), the ufe of which it is found fo difficult to prevent, while at the fame time their difeafes would be of a lefs malignant nature.

To prevent as much as poffible the ufe of that pernicious fpirit, fo deftructive to the health and morals of the men, let there be no houfe in the town licenfed to fell wine or brandy to the foldiers; and that a tax be laid on the retailers of the latter article, fo as to prevent the foldier from being able to purchafe it. Every regiment fhould have a canteen near the barracks, to fell nothing but wine, fubject to the infpection of an officer, to fee that it was of a good quality, and every precaution taken to prevent abufes.

When a fever of an infectious nature, has been introduced from fhips or generated in the barracks or hofpitals, (which frequently happens, when they are not fufficiently ventilated, and too much crouded), the moft effectual means fhould be employed to eradicate it, and prevent the infection fpreading. A ward fhould be particularly appropriated for the reception of thofe cafes: all their cloaths, bedding &c. fhould be completely fumigated, as well as their bodies, by a mode lately recommended by a very ingenious and eminent phyfician, Dr. Carmichael Smith, which is faid to have anfwered moft effectually in a highly infectious fever, which prevailed in fome prifons and fhips in England.

By attention to ventilation, cleanlinefs, proper diet, cloathing, bedding, and preventing the ufe of ardent fpirits, the moft beneficial and falutary effects would no doubt be produced, in a country where the climate is fo favourable to health, and I think is proved to have little or no fhare in occafioning the difeafes which are fo deftructive to the troops.

It has been often remarked, that among the numerous calamities attendant on warfare, infectious difeafe has ever been confidered the moft fatal foe: thofe who fall by the deftructive implements of war are few indeed, compared to the great number who perifh in our armies and fleets, by the fecret malignancy of this difeafe, as we find that no region of the globe is exempt from its baneful influence, where military operations are carried on. Every precaution we are acquainted with fhould be put in force to prevent the introduction of an enemy fo deftructive to the human race.

The of which, they are abundantly fupplied with vegetables, and every neceffary for preferving their health.—This falutary meafure, joined to the judicious regulations ordered to be purfued by Maj. Gen. Dundas, the Commander in Chief at the Cape, had a wonderful effect on the health of the army; for many months there was not one medical patient in the General Hofpital, for every phyfician there on the ftaff.

The bad construction of the hospitals, and want of proper arrangement in them, I consider a principal cause of the ill success which attends the military practice of physic, and to have greatly contributed to generate, and, I may say, nurse infection to that degree of virulence which has been fatally experienced in this and every other quarter, particularly in the West Indies, and on the continent; for a crouded hospital, badly situated, want of cleanliness, ventilation, and good nursing, will certainly counteract the best effect of medicine: this appears, by Dr. M'Lean, to have been severely felt, and fatally experienced, at St. Domingo.

With deference, I offer a sketch of two hospitals calculated for an army of six or seven thousand men, in this or any other healthy country; which I think, when assisted by great medical skill, would prove a formidable enemy to combat and destroy disease; and from particular enquiry, I can say, the expence of building them would be trifling considering their utility. Every choice of situation is to be had here (Cape of Good Hope), which is of the utmost importance in fixing an hospital.

We find the diseases which attend military service in every part of the world are, fever, dysentery, and ulcers; the latter, though not produced by contagion, yet from the fœtor, and putrid state of the sore, pollute the air, and render it in a great degree noxious to animal life. In the best ventilated hospital, where a number of morbid bodies are placed in one ward, foul air will be generated; this, joined to the distress both of body and mind, which each patient suffers from another, must aggravate every symptom of disease, and greatly retard the cure.

By each patient being placed in a separate appartment, many advantages would arise, tending to promote their recovery; infection would be destroyed, and entirely prevented; and the cure accelerated by rest and quietness.

The want of regular and orderly attendants on the sick, by employing men from the different regiments, besides weakening the military force, is found to be attended with other bad consequences to the service. I would therefore propose, that a corps be regularly appointed to the hospitals, under the name of hospital attendants; to be composed of sober and humane characters. The men to wear a uniform to distinguish them from the patients, and might consist of the following:—

*To the first Hospital, of* 120 *Patients.*

1 Ward-serjeant

1 Matron

1 Surgery-man

1 Barber

1 Cook

6 Washerwomen

20 Orderly-men

10 Nurses

4 Blacks, to keep the rooms and privies clean

—

45 Total.

To

*To the Convalescent Hospital, of 160 Patients.*

| | |
|---|---|
| 1 | Ward serjeant |
| 1 | Matron |
| 1 | Cook |
| 1 | Surgery-man |
| 1 | Barber |
| 6 | Washerwomen |
| 4 | Orderly-men |
| 4 | Nurses |
| 2 | Blacks, to keep the rooms clean |
| 21 | Total. |

STEWART HENDERSON.

*The following Outline of a Plan, for Preventing the Introduction of Infectious Disease into the Garrison at Chatham, was forwarded to the War-Office by* Mr. Wood, *Surgeon to the Garrison.*

Sir, *Upper Barracks, Chatham, May 1st,* 1791.

I beg leave to propose a plan which, I think, if adopted, would be the means of putting a stop to the introduction of the jail-fever into the garrison, and prevent the further progress of that malignant disease. It has one thing to recommend it, which perhaps may be deemed a consideration, viz. that it will put government to little expence, as well as prevent many desertions, and some diseases, the consequence of irregularities.

We know the causes of this disease to be foul air, and want of cleanliness; of course, the men most liable to it, are those confined in prisons, and other unventilated places, where cleanliness to their persons is not attended to; and what renders the introduction of this fatal disease the more to be dreaded is, that men of this description often retain the infection, and have the power of communicating it to others, though at the time, they have no symptoms of the disease; for it is known to lay dormant a considerable time, until brought into action by some exciting cause.

The garrison at Chatham being the general receptacle for recruits, many of them brought from the Savoy, and other prisons in England, it is evident what a source of infection must be introduced and propagated through the barracks by their mixing promiscuously; nor is it possible to eradicate it while the fresh seeds of infection are daily from all quarters brought in.

To obviate those evils, I would propose that a ship of the line (one of the old hulks) be employed as a lazaretto, or receiving ship, and stationed, for conveniency, opposite the garrison.

The recruits, instead of being brought into the barracks, to be sent immediately on their arrival, on board of this ship (particularly those who have been lately in prison), to be stripped of their filthy cloaths, which should be burnt, as well as every thing about them that appears to retain infection.

Their

### A Table calculated for Noſological and Meteorological Obſervations, in Hoſpitals, as well as in private Practice.

| STAGE FIRST. | STAGE SECOND. | STAGE THIRD. |
|---|---|---|
| *State of the Patient's Health, before he became ſubject to the Diſeaſe.* | *Commencement of the Diſeaſe, and its Symptoms, previous to its being ſubmitted to Medical Treatment.* | *The preſent State of the Diſeaſe, and its Progreſs.* |

| Determination of the Diſeaſe. | | | | | | | | | | Method of Cure indicated. | | | | | | | | Further Progreſs of the Diſorder. | | | | | | | |
|---|---|---|---|---|---|---|---|---|---|---|---|---|---|---|---|---|---|---|---|---|---|---|---|---|---|
| Days of the | | State of the head and organs of ſenſe. | Taſte. | Appetite | Thirſt | Tongue | Reſpiration | Expectoration | Hypochondriac and Abdominal Region | Stools. | Urine. | State of the Skin, of Heat or Cold, Perſpiration, Eruption and Colour. | Pulſe. | Nature of the Blood. | Sleep. | Particular Symptoms. | Evacuating Organs, by which Nature tends to accompliſh a Criſis. | Noſological Character of the Diſeaſe. | Medicines. | | Diet. | Daily State of the | | | |
| Month | Diſeaſe. | | | | | | | | | | | | | | | | | | Internal. | External. | | Barometer. | Thermometer. | Winds. | Weather. |
| | | | | | | | | | | | | | | | | | | | | | | | | | |

Farther Obſervations, chiefly reſulting from an accurate and regular uſe of the preceding Table.

| Termination of the Diſeaſe. | Influence of the prevailing Conſtitution of the Seaſon, on epidemic, ſporadic, acute, and chronic Diſeaſes, whether in the public Hoſpitals or not. | Appearance of the Organs after Diſſection. | Particular remarks on extraordinary Caſes. |
|---|---|---|---|
| | | | |

Their bodies to be well washed with warm water and vinegar, and the utmost cleanliness attended to while they remain on board.

That a superintending officer, with a surgeon, be appointed to give directions respecting the salutary regulations, and medical assistance, in such cases as may occur.

Proper non-commissioned officers, to be on board, for putting the regulations strictly in force, and preserving order and regularity.

The recruits to remain on board at least a fortnight; but if any appear suspicious, which may be judged from their unsalutary appearance, visible to every medical observer, let those remain a week longer. The itch, and other slight contagious diseases, may be cured before they are sent on shore.

That a ship of a smaller size be employed as an hospital, for the purpose of receiving such men/as may be attacked with the malignant fever; who should be removed from the receiving ship on the first appearance of the disease; and as the men can be mustered daily by an officer and surgeon present, the earliest attack may be discovered, which is of the utmost importance towards the cure of the disease.

Such is the outline of a plan which, I think, with some improvements, that you and others, more conversant in the service, are capable of making, would tend most effectually, not only to put a stop to the introduction, but check the progress of a disease, which proves so destructive to the troops.

I am, Sir,

Your most obedient, and

Humble Servant,

STEWART HENDERSON.

To VINCENT WOOD, Esq.

*Surgeon to the Forces, at Chatham.*

## Figures and Tables

**Figure f1:**
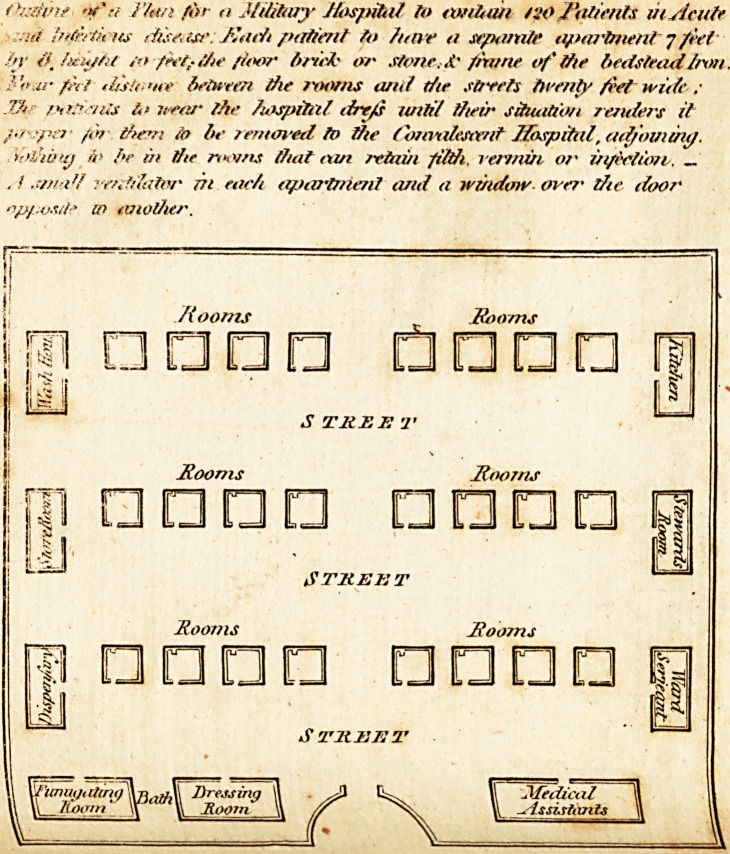


**Figure f2:**